# Metastatic Progression of an Aggressive Hepatoblastoma Involving the Left Atrium and Ventricle

**DOI:** 10.7759/cureus.39293

**Published:** 2023-05-21

**Authors:** Emre Yılmaz, Numan Kılıç, Ufuk Doğru

**Affiliations:** 1 Cardiology, Giresun University, Faculty of Medicine, Giresun, TUR

**Keywords:** cerebral metastasis, chemotherapy, left ventricular mass, left atrial mass, hepatoblastoma

## Abstract

Hepatoblastoma is the most common malignant liver tumor in early childhood. The metastatic extension of hepatoblastoma into the left atrium via the pulmonary vein and left ventricle is rare. Reported cases almost always involve right-sided approaches and occur in pediatric patients. However, we are reporting a case of a 22-year-old female with recurrent hepatoblastoma at multiple sites, including the left atrium, left ventricle, brain, and lung.

## Introduction

Hepatoblastoma is a rare tumor overall, but it is the most common primary malignant hepatic neoplasm in children less than five years of age, accounting for two-thirds of primary liver tumors in this population [1]. Over the years, different treatment protocols have successfully treated hepatoblastoma, increasing survival rates in some cases [2]. One of these treatment modalities is the sequential application of adjuvant chemotherapy and surgical resection. The survival rate is relatively high in cases where the tumor is resectable with chemotherapy, with a five-year overall survival rate of 75% in the SIOPEL-1 study [2].

Cardiac metastases of hepatoblastoma are extremely rare (0.67% to 3% of cases) [3,4]. Previously, metastases to the right atrium via the inferior vena cava, to the right ventricle associated with the pulmonary artery, and to the left atrium via the pulmonary vein have been reported [5-7].

However, no case report has described the recurrence of childhood hepatoblastoma with left atrial and ventricular involvement in adulthood. This report presents a patient with diffuse metastatic foci and left atrial and ventricular metastases. We discuss the status of the cardiac mass in this patient, who was deemed surgically inoperable after ongoing chemotherapy and completed radiotherapy courses.

## Case presentation

**Figure 1:**
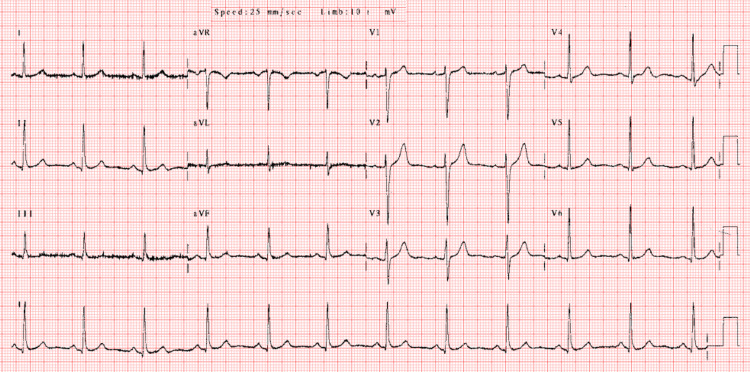
Twelve-lead electrocardiography image of the patient at emergency admission

A 22-year-old female patient presented to the emergency department with complaints of general malaise and shortness of breath. Our cardiology clinic evaluated her, where respiratory sounds were not heard in the upper left zone and were diminished in the bilateral lower zones, particularly with rales in the lower right zone, and no murmurs were heard. She was 37 kilograms and her body mass index was calculated as 13.3 kg/m^2^. The patient had bilateral peripheral edema (right leg ++/left leg ++) and a feeling of bloating in the abdomen. However, ascites was not detected in the abdomen on physical examination. Jugular venous distension was also not observed. There were no pathological features in other system examinations. The patient's vital signs were as follows: oxygen saturation (98%), temperature (37°C), blood pressure (110/60 mmHg), and pulse (75 beats per minute) measurements were within normal limits. Electrocardiography revealed no arrhythmia or ischemic changes (Figure 1). Laboratory test results are presented in Table 1. The patient's liver enzymes (alanine and aspartate aminotransferase) were borderline elevated. Brain natriuretic peptide levels were above the reference range and alpha-fetoprotein levels were within normal limits. There were no abnormalities in other laboratory tests.

**Table 1:** First admission laboratory results of the patient

	Patient’s results	Reference range
Hemoglobin (g/dL)	12	12-16
Creatinine (mg/dL)	0.5	0.5-1.1
C-reactive protein (mg/L)	6	0-5
Alanine aminotransferase (U/L)	48	0-33
Aspartate aminotransferase (U/L)	47	0-32
Albumin (g/L)	41	35-52
Total bilirubin (mg/dL)	0.8	0.3-1.0
Direct bilirubin (mg/dL)	0.15	0-0.2
Sodium (mEq/L)	139	136-145
Potassium (mEq/L)	4.2	3.5-5.1
Calcium (mEq/L)	9.1	8.6-10.2
Brain natriuretic peptide (pg/ml)	130	<100
Alpha-fetoprotein (ng/ml)	13	0-14

**Figure 2:**
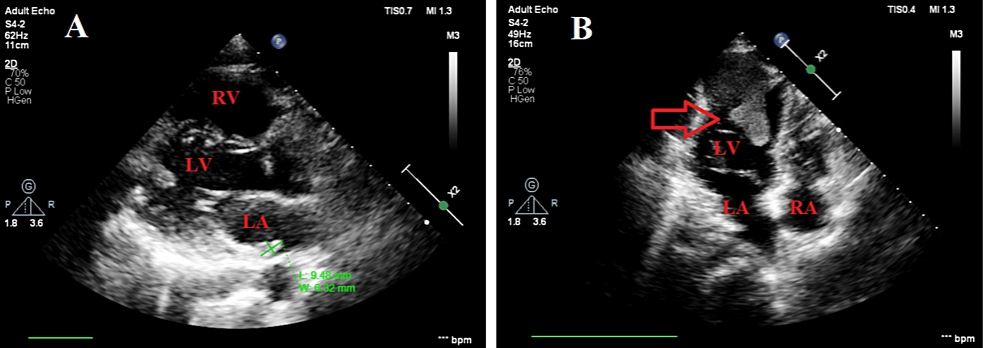
Transthoracic echocardiographic images of the left atrial and ventricular masses A: View of the left atrial mass in the parasternal long-axis window. B: Image of the left ventricular mass in the apical four-chamber window. LA: left atrium; LV: left ventricle; RV: right ventricle; red arrow: LV apicolateral mass.

In echocardiography, the ejection fraction was 60%, and we detected mild aortic insufficiency in the bicuspid aortic valve. We measured pulmonary arterial pressure as 30 mmHg, and right heart chambers and functions were within normal limits. We also observed a mass image measuring 41 x 39 mm at the left ventricle (LV) apicolateral, with trabeculations measuring 11 x 15 mm at the widest point. The LV mass did not negatively affect left ventricular hemodynamics or performance. We detected a mass image measuring 9 x 6 mm in the posterior of the left atrium (LA) and a 10 mm pericardial effusion that did not create compression at the broadest point adjacent to the LV lateral wall (Figure 2 and Video 1).

**Video 1:** Transthoracic echocardiographic video images of the left atrial and ventricular masses

**Figure 3:**
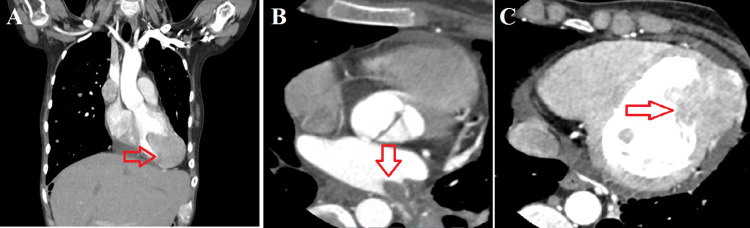
Contrast-enhanced cardiac computed tomography images of the left atrial and ventricular masses A: Coronal section view of the left ventricular mass (red arrow shows the left ventricular mass). B: Transverse section image of the left atrial mass (red arrow shows the left atrial mass). C: Transverse section image of the left ventricular mass (red arrow shows the left ventricular mass).

The patient's echocardiography findings were similar to previous measurements conducted in August 2022. There was no regression in the LV and LA masses. Compared with the cardiac computed tomography (CT) obtained in January 2022, which was the time of initial diagnosis, we observed the progression of the LV and LA masses. In the cardiac CT of January 2022, we observed a metastatic mass with polypoid extensions into the cavity on the left ventricular apical lateral wall measuring 39 x 38 mm and a tumoral thrombus extending to the LA by filling the left inferior pulmonary vein. The contrast-enhanced cardiac CT planned during hospitalization showed that the mass had transmural spread in the apical region and exhibited an extension of up to 50 mm toward the LV cavity from the apex at its widest point (Figure 3).

**Figure 4:**
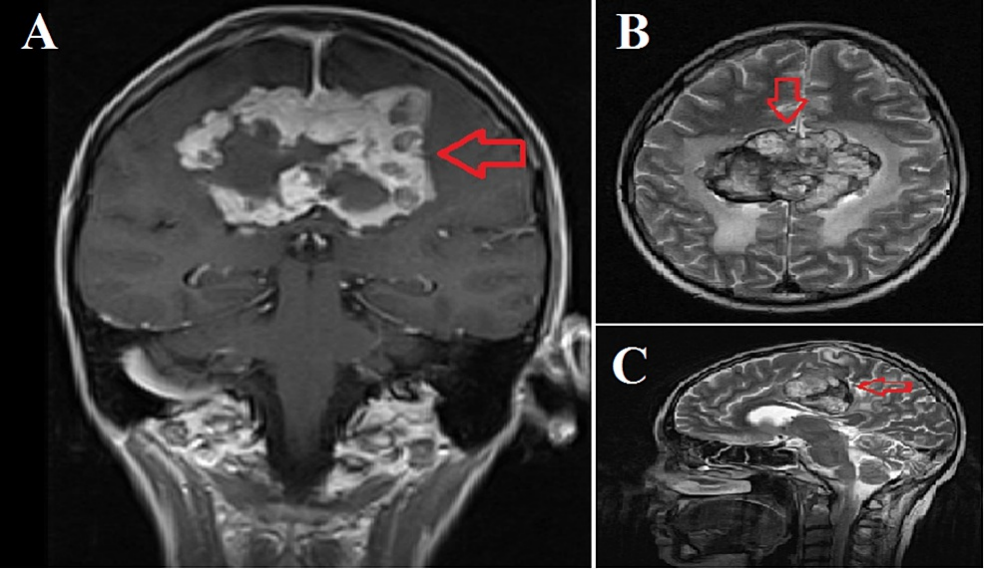
Magnetic resonance images of the cranial metastatic mass A: Coronal section view of the cranial metastatic mass. B: Transverse section view of the cranial metastatic mass. C: Sagittal section view of the cranial metastatic mass. Red arrow: the cranial metastatic mass.

The patient was diagnosed with hepatoblastoma and underwent right lobe liver resection at 11 years old in 2011. After six cycles of chemotherapy (cisplatin and doxorubicin: PLADO trial and SIOPEL protocol), she attained remission. In 2016, left upper lobe posterior segment tumor resection was performed due to the observation of lung metastasis, followed by nine cycles of chemotherapy. In 2021, after skin and brain metastases were detected, doxorubicin-carboplatin chemotherapy was administered for three months. Despite receiving regular chemotherapy and radiotherapy, a metastatic lesion was observed in the interhemispheric fissure, measuring 40 x 71 mm with lobulated contours, intense contrast enhancement, and hemorrhagic component on cranial MRI in February 2023, which showed progression compared to the earlier MRI in July 2022 (Figure 4).

**Figure 5:**
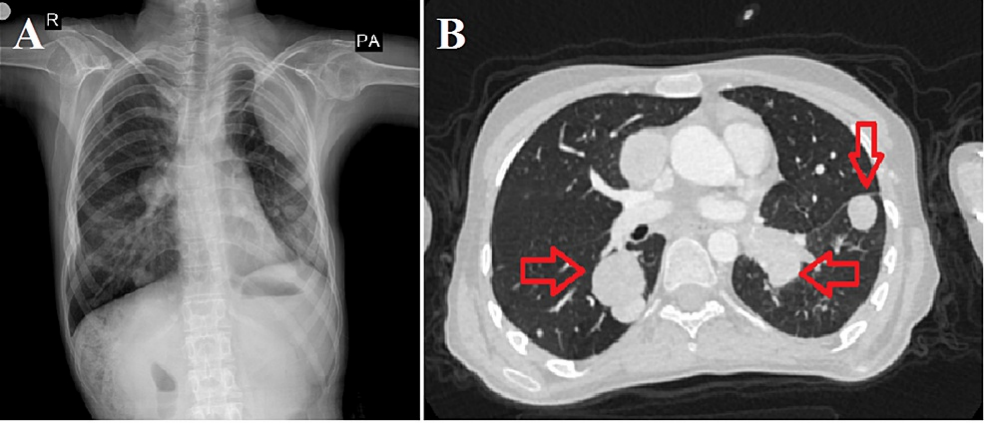
Posteroanterior chest X-ray and thorax computed tomography images of pulmonary metastatic nodules and foci A: Posteroanterior chest X-ray. B: Transverse section view of thorax computed tomography images of pulmonary metastatic nodules. Red arrow: pulmonary metastatic nodules.

In January 2023, metastatic lymph nodes were observed in the mediastinum, including a 30 x 24 mm mass in the right pericardiac area. Metastatic nodules were also observed in both lung parenchymas. The largest was a 52 x 45 mm mass in the left upper lobe, which had increased in number and size during the interval period. A metastatic lymph adenopathy (LAP) mass lesion measuring 24 x 28 mm was observed, obstructing the left lower lobar bronchus (Figure 5).

In January 2022, the heart team evaluated the patient due to cardiac metastases deemed inoperable due to the high mortality risk of possible cardiac tumor resection. The heart team suggested follow-up with chemotherapy and radiotherapy. Despite receiving 10 courses of radiotherapy and regular treatment with paclitaxel and carboplatin agents, the metastatic foci were widespread, and regression could not be achieved in these foci. We treated the patient with diuretics during hospitalization. The patient's peripheral edema and pulmonary congestion improved during the hospitalization, and the orthopnea and dyspnea subsided. Upon re-evaluation for cardiac mass surgery, the heart team recommended palliative care due to the widespread metastatic foci and spread of the cardiac mass. We decided on the follow-up of the cardiac mass with the continuation of chemotherapy and the patient was discharged. We planned diuretic treatment to prevent cardiac overload findings of our patient. We started low-dose anticoagulant therapy because cardiac masses may cause cardioembolic events. We could not plan aggressive medical treatments to prevent cardioembolic events because of the hemorrhagic component observed in the cranial mass in the control cranial MRI. Our patient, who was immobile due to cachexia and terminal malignancy, was followed up with low molecular weight heparin and was discharged.

## Discussion

We present this case report to emphasize the widespread metastases and recurrence with cardiac involvement of an aggressive hepatoblastoma case. We aimed to draw attention to the rare occurrence of childhood hepatoblastoma with widespread metastases and cardiac involvement in adulthood.

Reports on the metastatic spread of hepatoblastoma cases have been previously described in the literature. Especially in patients with cardiac involvement, metastases to the right atrium and right ventricle have frequently been localized and reported [5,6]. Even left atrial involvement through the pulmonary venous route has been reported, and management recommendations have been listed [7]. However, there is insufficient information regarding left ventricular involvement in the literature. Metastases can reach the heart through lymphatic or hematogenous routes or direct or transvenous spread. The lymphatic spread tends to result in pericardial metastases, while hematogenous spread preferably leads to myocardial metastases. [8]. We emphasize that in hepatoblastoma cases exhibiting hematogenous spread, LV may also be a potential site of metastasis.

Previous reports on hepatoblastoma have described cases where cardiac mass regression and cure were achieved with chemotherapy [5]. Some patients underwent simultaneous liver mass and cardiac metastasis resection, while others underwent isolated cardiac metastasis resection [6,7,9,10]. Algorithms for approaching metastatic masses of the left ventricle or other heart chambers have been reported in the literature [11]. However, when explicitly evaluated for hepatoblastoma, there is not enough treatment approach for left ventricular metastasis. Patients with multifocal involvement, venous invasion, distant metastasis, unresponsive to chemotherapy, and those who develop recurrence after surgery are complex patient groups to treat hepatoblastoma. It is difficult to talk about a standard treatment in these patient groups. Our case is challenging, with features such as distant metastases, recurring after primary tumor surgery, and inadequate response to chemotherapeutic agents. In addition, our patient's histological subtype of hepatoblastoma consisted of small-cell undifferentiated cells, and a low alpha-fetoprotein level may be the reason behind this aggressive course and resistance to treatment. Aggressive course and resistance to chemotherapeutic agents have been reported previously in patients with low alpha-fetoprotein levels [12]. Cardiac surgery was considered high-risk and inoperable due to widespread metastasis, high risk of mortality, and the difficulty of total excision of the cardiac mass. We planned the follow-up with chemotherapy and radiotherapy based on similar examples in the literature. Still, unfortunately, the treatment could not achieve the expected response in the size of the cardiac mass. The patient is still under palliative care and chemotherapy follow-up.

## Conclusions

Childhood hepatoblastoma may recur in adulthood with distant metastases. The brain, lung parenchyma, and cardiac cavities may be possible focal points for these metastases. Hepatoblastoma cases should not neglect the possibility of left ventricular involvement. Early, regular, and aggressive screenings are essential in hepatoblastoma cases. Despite regular chemotherapy and radiotherapy applications, regression may not be achieved in cardiac masses. Early detection of cardiac metastases can increase the success of treatment protocols.
